# Influence of Chabazite Zeolite Foliar Applications Used for Olive Fruit Fly Control on Volatile Organic Compound Emission, Photosynthesis, and Quality of Extra Virgin Olive Oil

**DOI:** 10.3390/plants13050698

**Published:** 2024-02-29

**Authors:** Lucia Morrone, Luisa Neri, Osvaldo Facini, Giulio Galamini, Giacomo Ferretti, Annalisa Rotondi

**Affiliations:** 1 Institute of BioEconomy, National Research Council, Via Gobetti 101, 40129 Bologna, Italy; luisa.neri@ibe.cnr.it (L.N.); osvaldo.facini@ibe.cnr.it (O.F.); annalisa.rotondi@ibe.cnr.it (A.R.); 2 Department of Chemical and Geological Sciences, University of Modena and Reggio Emilia, Street Giuseppe Campi, 103, 41125 Modena, Italy; giulio.galamini@unimore.it; 3 Department of Physics and Earth Sciences, University of Ferrara, Via Saragat 1, 44122 Ferrara, Italy; giacomo.ferretti@unife.it

**Keywords:** *Bactrocera oleae*, BVOCs (biogenic volatile organic compounds), olive oil, olive tree, photosynthetic rate, zeolites

## Abstract

The olive fruit fly (*Bactrocera oleae* Rossi) is the most dangerous pest of olive fruits and negatively influences the chemical and sensory quality of the oil produced. Organic farms have few tools against this pest and are constantly looking for effective and sustainable products such as geomaterials, i.e., zeolite. Since a particle film covers the canopy, a study was carried out on the olive tree’s responses to zeolite foliar coating. The tested treatments were natural zeolite (NZ), zeolite enriched with ammonium (EZ), and Spintor-Fly^®^ (SF). EZ was associated with higher photosynthetic activity with respect to the other treatments, while no differences were found between SF and NZ. Foliar treatments affect the amount of BVOC produced in both leaves and olives, where 26 and 23 different BVOCs (biogenic volatile organic compounds) were identified but not the type of compounds emitted. Foliar treatment with EZ significantly affected fruit size, and the olive fruit fly more frequently attacked the olives, while treatment with NZ had olives with similar size and attack as those treated with Spintor-Fly^®^; no difference in oil quantity was detected. Oil produced from olives treated with NZ presented higher values of phenolic content and intensities of bitterness and spiciness than oils from those treated with EZ and SF. According to the results of this study, using zeolite films on an olive tree canopy does not negatively influence plant physiology; it has an impact on BVOC emission and the chemical and sensory characteristics of the oil.

## 1. Introduction

The olive fruit fly (*Bactrocera oleae* Rossi) is the most harmful pest of olive fruits that strongly impairs the quality and quantity of olive oil, thus causing significant economic losses [1]. The larvae are monophagous on olive fruits. Females lay their eggs inside the fruit, and once hatched, the larvae feed on the fruit pulp. It is estimated that each larva consumes between 50 and 150 mg during the growth stage [2]. Oxidative and fermentation phenomena thus occur along the tunnel created due to hydrolytic and lipolytic enzymes, both endogenous and of bacteria and fungi, entering from the exit holes made by olive fruit flies [3]. This condition compromises the chemical and sensory quality of the oil [4].

Against *B. oleae*, management strategies can be (1) biological, such as a novel insecticide (es. Spintor-Fly^®^), mass trapping programs, sterile insect technique, particle film, and biological control using natural enemies, and (2) conventional, such as organophosphate (OP) insecticides (e.g., dimethoate) [1]. OPs are the most used pesticide in conventional olive groves; however, they are associated with negative environmental impacts and damage to the entomo-fauna [5], leading to insecticide resistance [1]. The olive fruit fly infestation management in organic farming is based on less-damaging strategies such as using the least-harmful insecticides (Spintor-Fly^®^), mass trapping programs, and particle film applications [1]. This last technology has gained more interest as a valid and eco-compatible alternative to chemical pesticides. Besides kaolin, natural zeolites can also be applied in particle film technology. Foliar treatments based on zeolites are less known and used than kaolin for their more recent application in the agricultural sector. Zeolites are a family of aluminosilicate minerals composed of 3D frameworks of linked [SiO_4_]^4^- and [AlO_4_]^5^- tetrahedra that delimit microporous structures of channels and cages with high specific surface areas and cation exchange capacities. Synthetic and natural zeolites have been widely applied for their capacity to adsorb and separate cationic species, reducing the environmental impacts of wastes while recovering important elements, such as ammoniacal nitrogen [6,7,8,9]. In this optic, zeolites can be used to vehiculate essential nutrients to plants via foliar application [10,11,12].

Nitrogen is the mineral nutrient most widely used in olive growing; it affects plant growth [13] and, therefore, photosynthesis [14] since a significant part of the foliar N is used in photosynthetic apparatus [15]. A foliar nitrogen supply can be useful to supplement ordinary soil fertilization when root uptake is poor due to the absence of rainfall [16]. The use of zeolites modified with ammonium ions could bring synergic effects for plant health, as the nutrient could be preferentially absorbed into the leaf tissues.

Zeolite foliar treatments showed interesting results on grapevines for containing fungal diseases [17], on apple trees [18], and olive trees in conditions of water stress [19]. A combination of organic biostimulants with an inorganic corroborant (zeolite) was also tested for the plant response to the quarantine pest tomato leaf curl New Delhi virus [20].

*Olea europaea* L. is a low-emitting species with no or very low biogenic volatile organic compound (BVOC) emissions, and studies on its BVOC are still scarce [21,22,23,24,25]. BVOCs include compounds of diverse chemical classes, such as isoprene, terpenes, alkanes, alkenes, alcohols, esters, carbonyls, and acids, and different physiological processes in many plant tissues produce them. BVOC emission can be constitutive or induced. Constitutive emissions can be observed throughout the life cycle of a plant or, more often, at specific developmental stages (such as leaf and needle maturation, senescence, flowering, and fruit ripening) [26] and are used to “communicate” with the environment, e.g., to attract pollinators and herbivore predators or as deterrents against pathogens and herbivores [27]. Induced BVOC production and emission are usually in response to environmental drivers, such as both biotic or abiotic stress, to confer protection [27] and improve the plant’s resilience. Environmental stresses may induce a change in constitutive BVOC, either stimulating or quenching the emissions or inducing de novo synthesis and emission of BVOC, even in a systemic way, thus away from the site of damage [26]. Increased BVOC emissions were registered from leaves as a response to environmental stresses [28], herbivore infestation, and mechanical damage [25]. In olive trees, BVOC emissions from leaves and fruits are also influenced by the phenological phase and ripening degree [29]. The importance of BVOC in olive trees in the attraction or repulsion of pathogens has long been known; the first work on the olive cultivar Itrana was carried out in 1993 [29], identifying some BVOC (e.g., α-pinene) as attractive, and others (e.g., trans-2-hexenal) as repellents for the oviposition of the olive fly. A few studies have been carried out on the relationships between BVOC emission and *B. oleae* infestation, clarifying that *B. oleae* displays oviposition preference for some olive cultivars and also for the ripening stage since females generally prefer to oviposit on unripe green olives [30]. To successfully locate and select oviposition sites, the adult females of *B. oleae* mainly rely on olfactory cues related to specific BVOCs emitted by olive trees, such as α-copaene and toluene, which could act as oviposition promoters [21,22,30].

The use of geomaterials such as zeolite against the olive fly is increasingly established among olive growers, who are looking for eco-sustainable methods to respond to the market demand for sustainable products. This study aims to evaluate how the presence of zeolite particle films could influence BVOC emissions, ecophysiological parameters, and the chemical and sensory characteristics of the olive oil produced.

## 2. Results

### 2.1. Photosynthetic Rate and Leaf Analyses

The photosynthetic rate of leaves (A) is shown in Table 1; all treatments tested exhibited similar trends, undergoing a slowdown in photosynthesis in the early days of September, as temperatures were high, and then rising again on subsequent dates. Considering that the treatment with SF requires a localized application in only some plants and that these points have been discarded for photosynthesis measurements, this treatment can be considered a negative control since the remaining part of the canopy has not undergone any covering. No significant differences were found between NZ and SF; moreover, photosynthetic activity was even higher in EZ than in the other two treatments. However, no differences were found in the leaf content of C and N (Table 2), but a higher content of δ15N was recorded in leaves treated with enriched zeolite. A complete element characterization of leaves is reported in Supplementary Table S2.

### 2.2. BVOC Emissions on Leaves and Fruits

#### 2.2.1. Leaves

From leaves emissions, 26 compounds were identified, belonging to different chemical classes (alcohol, aldehyde, alkane, arene, ketone, aromatic compound, and phenol) (Table 3), with total emission fluxes of around 3 ng m^−2^ s^−1^, regardless of treatment. The most abundant compounds released by leaves were 2-ethylhexanol, nonanal and decanal, toluene, 1-hydroxycumene, and alkanes, such as undecane. To the authors’ knowledge, no BVOC profiling has been previously reported for the Correggiolo cultivar. Compounds identified in this experiment, such as the aldehydes benzaldehyde, nonanal and decanal, and the cumene o-hydroxy cumene, were part of the profile of different *O. europaea* cultivars; however, the Correggiolo variety presents a characteristic emission profile, different from the other cultivars tested so far. The foliar treatments significantly affected BVOC emission composition from leaves; in detail, EZ treatment increased toluene emissions, while ethylbenzene, o- and p-xylene, acetophenone, and hydroxy-cumene emissions were higher in both the NZ and EZ compared to the control (SF), decane emissions were higher in NZ, and benzonitrile emissions were higher in the control. However, in our experiment, α-copaene was not recorded in leaves, while toluene emissions were higher in EZ, the treatment with the highest photosynthetic rate and the most affected by *B. oleae*.

#### 2.2.2. Olives

In BVOC emission from olives, 23 compounds were identified, belonging to different chemical classes (alcohol, aldehyde, alkane, arene, ester, ether, ketones, monoterpenoids, sesquiterpenes, furan, aromatic compounds, and phenol) (Table 4). The most abundant compounds released by fruits were 2-propanol, ethyl ether, 2-propanone, octane, and tetrahydrofuran. The foliar treatments and collection times affected BVOC emission composition from drupes (Table 3); in detail, EZ treatment increased hexanal and nonanal emissions, but only during the second collection, while 2-propanol emissions were higher in SF treatment, only during the second collection, benzene emissions were higher at the second collection time for both EZ and SF, while undecane emissions were higher during the first collection. 2-pentanone and the furane tetrahydrofuran emissions were higher in control SF during the second collection, while the ketone 2-propanone emissions were higher in SF as well, but only at the first collection. *Cis*-3-hexenyl acetate and octanal emissions, albeit very small, varied according to the treatment and collection times. The putative oviposition promoters to olive fly α-copaene and toluene were released from fruits at both collection times; the content of these compounds did not seem influenced by the treatment or the fruit ripening.

### 2.3. Fruit Analysis

Regarding ripeness, treatments did not influence the ripening of olive fruits. At the harvest time, the RI of the EZ treatment was 1.35, 1.4 for NZ, and 1.5 for SF.

**Figure 1:**
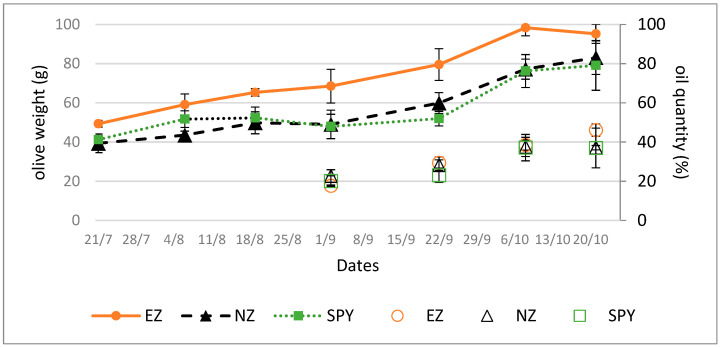
Olive weights (line) and oil content (dots, % on dry weight) in olives from plants submitted to tested treatments of natural zeolite (NZ), zeolite enriched with ammonium (EZ), and Spintor-Fly^®^ (SF). Data are expressed as the mean of three replicates ± standard deviation.

Plants treated with EZ produced larger olives compared to those treated with SF or NZ, as suggested by olive growth trends (Figure 1, line).

The trend in oil accumulation, as shown in Figure 1 (dots), was not affected by the treatments studied.

**Figure 2:**
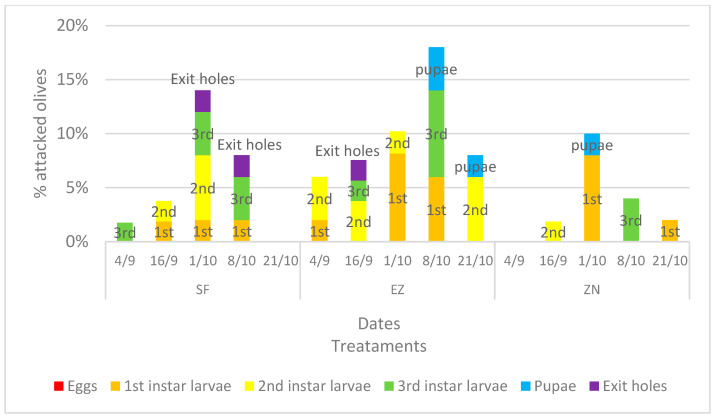
Percentage, divided into different larval stages (eggs, 1st, 2nd and 3rd instar larvae, pupae and exit holes), of attacked olive fruits developed under different treatments: natural zeolite (NZ), zeolite enriched with ammonium (EZ), and Spintor-Fly^®^ (SF).

The incidence of olive fruit fly infestation is shown in Figure 2. Growth in the infestation can be seen during the first three survey dates; however, from the chi-square analysis on the number of olives attacked, the treatments under study did not differ. The data of attacked olives from the last two dates exhibited a slight significance (*p*-value < 90%), namely *p* < 0.056 on the date of 8 October and *p* < 0.068 on the date of 21 October.

### 2.4. Climatic, Soil Data, and Flight Curve

**Figure 3:**
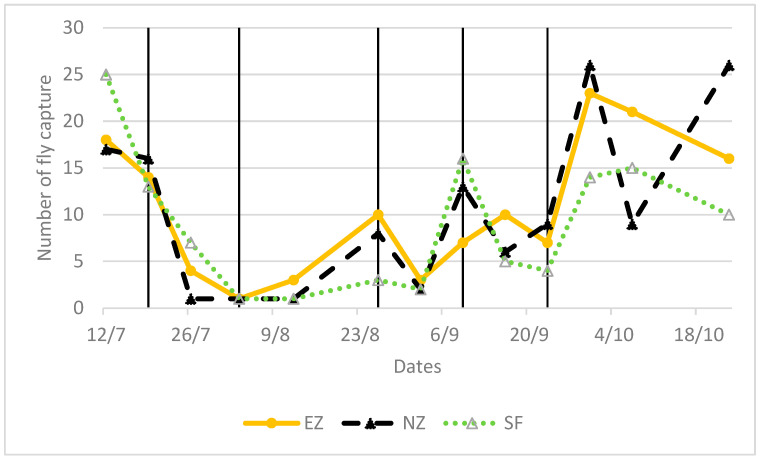
Flight pattern of *Bactrocera oleae* in the tested treatments of natural zeolite (NZ), zeolite enriched with ammonium (EZ), and Spintor-Fly^®^ (SF). Vertical line indicates the dates of foliar treatments.

**Figure 4:**
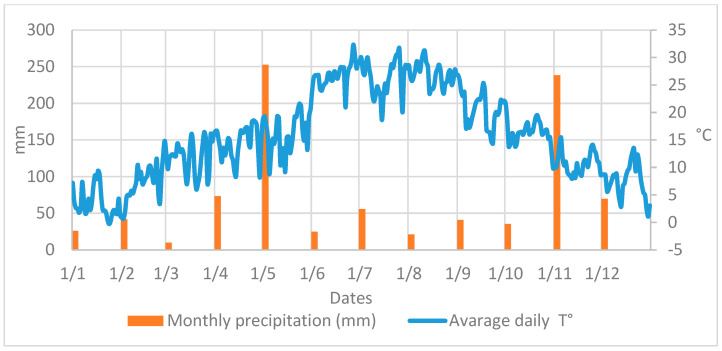
Atmospheric temperature and rainfall data collected in area of study.

Both NZ and EZ treatments did not show differences in the *B. oleae* fly curve with respect to conventional SF control. During the period of olive fruit ripening, a decrease in the number of adult flies is shown in Figure 3, from the end of June until August, likely related to the high atmospheric temperature and the scarce precipitations that occurred in the experimental area (Figure 4). Alternating increased and decreased trends of *B. oleae* adult flies, which were observed in all theses (NZ, EZ, and SF), can be explained by the periodicity of treatment applications, and these fluctuations increased at the end of September when more favorable climatic conditions contributed to the increase in fly captures.

According to the international USDA soil texture classification, the soil was a sandy loam with the following particle size composition (mean ± SD), without significative differences among the experimental thesis: sand (61.2 ± 7.7%), silt (29.7 ± 5.1), and clay (9.05 ± 2.78). The complete chemical analyses of soil are reported in Supplementary Material Table S1.

### 2.5. Chemical and Sensory Analyses of Olive Oils

Chemical analyses conducted on oils proven from olives that had undergone the different treatments under study showed no differences in acidity, number of peroxides, UV indices, and fatty acid profile (Table 5). Free acidity exhibited low values in all treatments, although the levels of olive infestation were different.

The oils presented a low peroxide value, whereas the oil obtained by plants treated with NZ exhibited the lowest value, probably due to the lowest level of infestation in olives. Oil produced by EZ and SF exhibited higher PV values with respect to NZ,

The absence of exit holes in NZ fruits was plausibly related to decreased oxidative processes compared to EZ and SF. This was likely reflected in the lower extinction coefficient at 232 nm (K232) recorded in the NZ oil, suggesting higher product quality.

**Figure 5:**
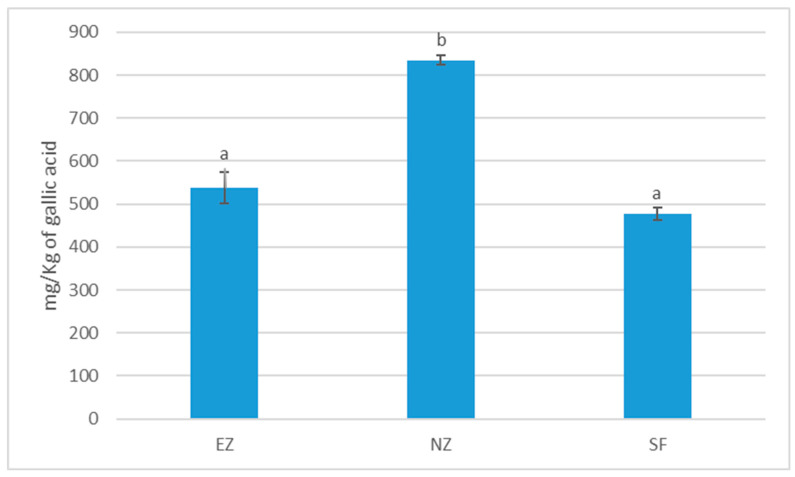
Total phenol content of oil samples cv. Correggiolo submitted to tested treatments of natural zeolite (NZ), zeolite enriched with ammonium (EZ), and Spintor-Fly^®^ (SF). Different letters above chart bars indicate significant differences (*p* < 0.05) according to the HSD test.

Treatment with NZ showed significantly higher total phenol content in olive oil with respect to EZ and SF (Figure 5).

The foliar treatments with EZ and SF have produced oils with similar sensorial profiles, while oil produced by plants treated with NZ exhibited statistically higher levels of some positive attributes like bitterness, pungentness, and grass (Table 6). Oils produced by NZ treatment also showed the highest level of grass attributes (Table 6). In this study, a lower ripening index of milled olives and a lower percentage of fly attacks (lower 20%) meant that no defects were detected in oils from all the treatments. In summary, the oil obtained from the plants treated with NZ was perceived to have a richer profile compared to the ones obtained with EZ and SF treatments. However, it is impossible to discern if this was due to the foliar treatment with NZ or to lower infestation levels and olive damage. 

## 3. Discussion 

It was hypothesized that the zeolite coating would cover the leaf in such a way as to hinder photosynthesis and thus disrupt light radiation absorption; instead, this did not occur, as no significant differences were found between NZ and SF in agreement with previous results found on the same Correggiolo cultivar. Moreover, a higher rate of photosynthesis was found in the treated plants than in the other two treatments. This could indicate that there is nitrogen release at the leaf level that the plant assimilates and uses in photosynthetic systems since more than half of the total leaf N is allocated to the photosynthetic apparatus [15]. However, in C and N leaves content, no differences were highlighted. These results agree with other works [14,15] on olive trees that showed reduced photosynthetic rates in leaves with a limited supply of N. 

*O. europaea* is a low BVOC emitter [21,23], which was confirmed by our study. BVOC emitted from leaves can act as an ovideposition activant or deterrent for *B. oleae*. Moreover, *O. europaea* cultivars can be distinguished based on their volatile fraction composition in leaves, as carried out by different authors [23,31]. In fact, leaves of different cultivars were found to release different compounds at different concentrations, i.e., α-farnesene, kongol, theaspiranes, and β-damascenone in cv. Frantoio, kongol, α-farnesene, nonanal and β-damascone in cv. Leccino, nonanal, kongol, β-damascenone, and β-damascone in cv. Cipressino [21], (*E*)-2-hexenal, (*E*,*Z*)-2,4-hexadienal, and (*E*,*E*)-2,4-hexadienal in cv. Olivastra Seggianese [31], while the most abundant in cv. Chemlali were (*E*)-2-hexenal, nonanal, (*E*)-β-damascenone, 3-ethenylpyridine, and caryophyllene [32]. Moreover, other compounds were identified as components of the BVOC profile of *O. europaea* leaves (cultivar not specified), such as styrene, xylene, octanal, nonanal, and -α-farnesene [21]. As detailed in the results section, we characterized for the first time the BVOC profile of leaves of Correggiolo cv., consisting mainly of 2-ethylhexanol, nonanal and decanal, toluene, 1-hydroxycumene, and alkanes such as undecane. According to Malheiro et al. [33], toluene and α-copaene released from leaves act as oviposition promoters to olive fruit flies; toluene can be induced in response to environmental stress [34] and is also correlated with infestation of different olive flies [4,21,29]. Our results support this thesis since toluene emissions were higher in the EZ treatment, which was also the most infested one, while α-copaene was not recorded in leaves, and maybe this could be a cultivar effect. 

BVOC emission from olives was also limited compared to other fruits, as previously reported for *O. europaea* [4,35]. *O. europaea* cultivars can also be distinguished on the basis of their volatile fraction composition in fruits [30,31], and as for leaves, the BVOC profile of Correggiolo fruits is different from the profiles identified in other cultivars, with the most abundant compounds in the analyzed drupes being 2-propanol, ethyl ether, 2-propanone, octane, and tetrahydrofuran. The emission of several BVOCs, such as the aldehydes hexanal, octanal and nonanal, the arene benzene, the alkane undecane, the ester *cis*-3-hexenyl acetate, the alcohol 2-propanol, the ketones 2-pentanone and 2-propanone, and the furane tetrahydrofuran, varied according to the different treatments. Emissions of saturated aldehydes such as hexanal, heptanal, octanal, nonanal, and decanal were observed from different plant species in response to both abiotic and biotic stress such as ozone exposure and pathogen and insect attacks [36]; their role is still unknown, but they were also found to be part of a bouquet perceived by a pest of almond trees [37]. As mentioned above, benzene can be released for leaf emissions as a response to multiple biogenic stresses [38], while undecane emissions might have a role in herbivore–plant interactions, as observed in tomatoes [39]. Cis-3-hexenyl acetate is among the typical BVOCs induced, locally but also systemically, following wounding by herbivores, for instance, in sweet potatoes [40]. Up to date, no papers have been published on the emission of 2-propanol, 2-pentanone, tetrahydrofuran, and 2-propanone from plants, even if the latter seems to be emitted from fungi in mixtures with antifungal properties [41]. As clear from the results above, plants in general and *O. europaea*, in particular, are not characterized by a single volatile but by a specific blend that can change according to the phenological phase, season, climatic conditions, presence of stressors and adaptation to them, and organ considered [25]. It is a complex scenario, and changes in specific ratios of the blend are more common and possibly more effective than changes in a single volatile. 

EZ foliar treatments could act as a nitrogen fertilization for the plant. In other species, N fertilization applied to soil was found to enhance BVOC emissions from leaves, mainly by promoting the leaf area index [42]. The literature on the effects of leaf nitrogen fertilization on BVOC emission is scarce, but some evidence exists for other plant species that influence BVOC emission [43]. The fertilization effect of EZ foliar treatment is shown in fruit analysis results since olives from EZ treatment were bigger than olives from the other treatments, in agreement with Proietti and colleagues [44]. However, no effect was observed on oil accumulation in olives during ripeness, which is consistent with Regni and Proietti’s [45] suggestion that the absence of an effect on oil accumulation may be attributed to a non-deficient nitrogen condition, and this probably explains the different results we obtained on the same cultivar but in a different location [46]. 

Angerosa et al. [47] reported that the extent of the decrease in oil quality also depends on the type of infestation; a key role is played by the presence of exit holes produced by larvae, which destroy the cellular integrity and expose the fruit’s inner tissues to oxygen, thus accelerating the hydrolytic and oxidative processes. It is interesting to underline that exit holes have never been observed in the olives treated with NZ, suggesting the death of olive flies before reaching the pupae stage. A possible explanation could rely on the effects that rock powders and, in particular, zeolite particle films can exhibit on modifying the bacterial flora present in olive leaves and fruits, including the endosymbiont *Candidatus Erwinia dacicola*, which is crucial for the development of *B. oleae* larvae in unripe olives [48]. Exit holes were observed only on the 16 September in olives treated with EZ (1.89%), while a percentage of 2% was recorded in the two surveys conducted in October in the SF, where exit holes were also observed. It is known that the fly attack is influenced by various factors, including the intrinsic susceptibility of the cv. In this study, a cv. classified with low susceptibility to olive fly was chosen; the Correggiolo cv., in fact, belongs to the cv. Frantoio group. A second factor that strongly influences the fly’s choices is the fruit size: this could explain the greater infestation observed on olives treated with EZ. In fact, during the middle samplings, EZ plants presented a statistically significant higher photosynthetic rate, which determined a greater growth and a greater weight of the fruits; therefore, a higher preference for the *B. oleae* female. 

The content of free acids in oils is one of the criteria for the classification of olive oil at various commercial grades, together with the peroxide number and spectrophotometric indices (K232, K 270). No differences in quality index values were highlighted for oils obtained from plants treated with NZ, EZ, and SF, which is in agreement with other studies conducted on kaolin [49] and zeolite [46]. As far as regards phenol content in olive oil, we found a higher content in NZ oil than the oil of the other two treatments; this result is in accordance with the observations of Rotondi et al. [46] that compared natural zeolite with kaolin in Correggiolo cv. Very few studies reported the effect of foliar zeolite treatments on the phenolic content of produced oils also because it is difficult to understand whether the content in phenolic substances is influenced only by the attack of the olive fruit fly or is also due to a plant ecophysiological response to the particle film covering the leaves and fruits [46]. However, the phenolic content in olive oils is an important parameter as it is associated with health benefits, extending product shelf life and sensory characteristics. In fact, NZ exhibited a higher total content, responsible for the bitter and pungent positive attributes of olive oils, in agreement with other reports [50]. 

## 4. Materials and Methods 

### 4.1. Field Site and Treatments 

**Figure 6:**
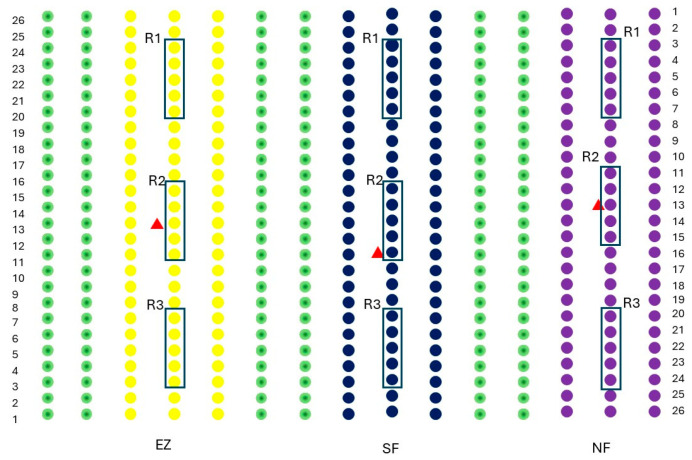
The experimental design adopted in this study. Purple dots indicate olives treated with natural zeolite (NZ), blue dots indicate Spintor Fly^®^ thesis (SF), yellow dots indicate zeolite enriched with ammonium (EZ) thesis and green dots are olive rows in between the studied treatments. Black boxes R1, R2, and R3 indicate the randomized groups per thesis, while the red triangle indicates the yellow sticky traps baited with the synthetic sex pheromone.

The study was carried out on a commercial olive grove (*Olea europaea*) located in San Lazzaro di Savena (44°27′00″ N, 11°23′33″ E; Emilia Romagna, Italy; 52 m s.l.s). The olive grove in which the study was conducted consisted of 15 rows of 26 trees each, with a planting spacing of 5 m × 4 m. The experiments were conducted on the Correggiolo cv. The tested treatments were natural zeolite (NZ), zeolite enriched with ammonium (EZ), and Spintor-Fly^®^ as conventional control (SF), and a protein bait with added spinosad, which is commonly used in organic farms for contrasting *B. oleae* infestation. The theses comprised three rows of olive trees, and three randomized groups (R1, R2, and R3) per thesis (NZ, EZ, and SF), each constituted by five olive trees, were considered for physiological measurements and fruit sampling (Figure 6). 

The natural zeolite (NZ) was supplied by Balco s.p.a company (Sassuolo, Italy). Its mineralogical composition is reported by Galamini et al. [51], while its affinity for NH_4_^+^ adsorption has already been investigated [8]. The zeolite used in the EZ treatment was previously modified with NH_4_^+^ prepared in accordance with the following procedure. The material was firstly washed with Milli-Q water and dried at 105 °C (48 h). An adsorption batch with 1 M NH_4_Cl was then performed with a solid-to-liquid rate of 25% (*w*:*v*). The mixture was stirred with an orbital shaker for 6 h at 170 rpm and 20 °C. The zeolite was then separated by centrifugation (4000 rpm for 5 min) and washed with Milli-Q water multiple times. The collected NH_4_^+^-modified zeolite was dried at a low temperature (60 °C for 48 h) to avoid any possible loss of nitrogen. 

Each treatment comprised 0.8 kg of natural zeolite per 100 L of water (NZ) and 0.4 kg of natural zeolite + 0.4 kg of NH_4_^+^-modified zeolite per 100 L of water (EZ). Both NZ and EZ treatments were applied in the aerial part of the groves using a portable sprayer with a flow max of 50 L/min and a capacity of 300 L (G.R. Gamberini, Bologna, Italy). Spintor Fly^®^ (SF) was applied according to the guidelines provided by the producer (Dow AgroScience, Milano, Italy). Foliar applications of treatments (NZ, EZ, and SF) started at the end of June, at the phenological stage of the onset of fruit susceptibility to *B. oleae* attacks. Applications were repeated every 20 days, with a total of six applications until harvest (17 July 2019, 31 July 2019, 24 August 2019, 9 September 2019, 27 September 2019, and 5 October 2019). All the experimental theses were managed under conventional orchard agronomic practices: soil fertilization, pruning, and winter treatment based on the Bordeaux mixture.

### 4.2. Climatic, Soil Data, and Flight Curve 

Air temperature was monitored with a sampling rate of 1 min using a Tinytag Ultra 2—TGU-4017 datalogger (Gemini Data Loggers, Chichester, UK), while rainfalls were monitored using a manual pluviometer (Raig, Barcelona, Spain). The soil analyses were collected according to Medoro et al., 2022 [52] to determine N and C content and the geochemical composition, respectively. 

The flight curves of adult *B. oleae* were monitored using yellow sticky traps baited with the synthetic sex pheromone. One trap for each thesis was installed in the middle row of each thesis from the beginning of July until the end of October, corresponding to the olive harvest. Traps were inspected every 7 days, and captured flies were counted and removed. The traps were changed every four weeks. 

### 4.3. Photosynthetic Rate and Leaf Analyses 

Photosynthesis (A), was measured during a clear sky using a Li-Cor portable photosynthesis system (LiCor 6400, Lincoln, NE, USA) operating at a 400 µmol m^−2^ s^−1^ flow rate. Measurements were taken in the morning (from 10:00 to 12:00 a.m.) according to protocols [53,54]. A total of twenty-four (eight for each randomized block) undamaged and mature sun leaves were selected for the measurements for each treatment, according to Larbi [55]. The total nitrogen and carbon (respectively, TN and TC) and the respective isotopic signature (δ15N and δ13C) of leaf samples were acquired with a Vario Micro Cube Elemental Analyser (EA) (Elementar, Langenselbold, Germany) connected to an Isoprime 100 Isotope Ratio Mass spectrometer (IRMS) (Isoprime, Cheadle, UK) operating in a continuous-flow mode. The EA-IRMS was calibrated with synthetic sulfanilamide (provided by Isoprime Ltd.) and Carrara Marble (cross-calibrated at the Institute of Geoscience and Georesources of the National Council of Researches of Pisa) standards [52]. 

### 4.4. BVOC Emissions on Leaves and Fruits 

BVOC emissions from leaves were sampled in mid-September to avoid any possible effect due to heat or drought stress, following the methodology described by Yuan [56]. Briefly, three biological replicates were considered per thesis, each constituted by a single leaf from three different plants. In detail, the central part of the leaf was included in a 3 cm^2^ cuvette of a portable infrared analyzer (Li-Cor 6400, Lincoln, NE, USA), setting the experimental conditions at a 30 °C temperature and 1000 µmol m^−2^ s^−1^ of PAR (photosynthetically active radiation). When photosynthesis reached a steady state, 4 L air samples were collected through a purified Tenax and Carbograph tube (Gerstel, Mülheim an der Ruhr, Germany) with a sampling pump (VSS-1, AP Buck, Orlando, FL, USA). Blank (leafless) samples were collected at the beginning and end of the sampling set. Traps were sealed with Teflon-coated brass caps immediately after collection and stored at −20 °C for analysis. 

BVOC emissions from olives were sampled in the lab with a dynamic headspace analysis. Batches of 60 g of fruits were randomly collected on the 10th of September, and after 10 days, before fruit veraison began, from five plants per thesis. Healthy fruits were selected and equilibrated overnight at room temperature. After equilibration, they were transferred into 100 mL glass jars where VOC-free air—generated by a homemade zero air generator—was flowed at a rate of 150 mL min^−1^ through a Teflon tubing connected to a gas inlet at 1 cm from the top of the extraction vessel. To trap BVOC produced by the fruit samples, the air exiting the flask was pulled from the outlet of the extraction system through steel tubes packed with Tenax TA 35/60, Carbograph 1 TD 40/60 ^®^ (Markes International, Ltd., Llantrisant, UK) connected to an external pump (Pocket Pump SKC Inc., Eighty Four, PA, USA) set at a flow rate of 200 mL min^−1^ over a 1 h period. Jars without olive samples were used as experimental controls (blanks) to determine background levels of VOC contamination. Traps were stored at −20 °C before analysis. 

A BVOC analysis was carried out, according to Baraldi [57]. In detail, BVOCs were released from traps using a thermal-desorption unity series 2 (Markes International, Sacramento, CA, USA) and injected into a 60 m capillary column internally coated with a 0.25 μm film of polymethylsiloxane (HP-1, 0.25 mm I.D., J&W Scientific USA, Agilent Technologies, Palo Alto, CA, USA). BVOC separation was performed on a 7890A gas chromatograph, and eluted compounds were measured with a 5975C mass detector (GC–MS, Agilent Technologies, Wilmington, NC, USA). Thermal desorption of the sampling tubes was carried out for 15 min at 280 °C with a helium flow rate of 50 mL min^−1^. The cold trap was then rapidly heated from 30 °C to 280 °C, and analytes were injected into the capillary column via a transfer line heated at 280 °C. Identified compounds were quantified using the external standard calibration procedure. BVOC emissions were expressed as ng m^−2^ s^−1^ for leaves and ng kg^−1^ min^−1^ for fruits. 

### 4.5. Fruit Analysis 

Fruit fresh weights, ripening index (RI), olive infestation levels, and oil accumulation trends were monitored in samples consisting of 100 olives per thesis, collected every 7 days, from the beginning of September to the harvest. The RI was calculated by dividing the drupes into seven classes according to different skin and pulp pigmentation [58]. In order to collect the infestation percentage, olives were externally and internally observed with a stereo optical microscope (Wild MZ-8 Leica, Wetzlar, Germany) and the number of immature stages, eggs, young larvae that included the first-instar larvae (L1) and the second-instar larvae (L2), mature larvae (third-instar larvae (L3), pupae, and exit holes were recorded [59]. 

Oil fruit contents were determined in three replicates using the gravimetric approach after oil chemical extraction using n-hexane on milled olive paste (IKA MF 10 basic Microfine grinder drive, Breisgau, Germany) and solvent evaporation under vacuum conditions [60]. Data were expressed as oil percentage per dry olive fruit weight. 

### 4.6. Olive Processing and Olive Analysis 

Olive samples were obtained by harvesting five plants for each thesis. Olives, processed within 24 h from the harvest, were defoliated, washed, and milled using a low-scale continuous mill (Oliomio^®^; Toscana Enologica Mori, Firenze, Italy) equipped with a blade crusher, a horizontal malaxator, and a two-phase decanter. For each sample, the technological settings of temperature (below 27 °C), time of malaxation (20 min), speed of the decanter (4200 rpm), and flux of water in the separator (0.8 L h^−1^) were standardized for minimizing variability due to the extraction procedures. Oil samples were filtered through cotton filters, poured into dark glass bottles, keeping the headspace to a minimum, and stored in a temperature-controlled cupboard set at 15 ± 1 °C until analysis.

Free acidity, peroxide value, UV-spectrophotometric indices (K232 and K270), and fatty acids were evaluated following the official methodologies (Regulation EC 2568/91 and subsequential modification) [61]. Fatty acids were analyzed using the gas chromatographic technique, using a Chrompack CP 9000 gas chromatograph with a flame ionization detector equipped with a capillary column (Stabilwax, Restek Corporation, Bellefonte, PA, USA) and helium as the carrier gas (flow rate = 1 mL min^−1^; split ratio = 1:20, *v*:*v*). Chromatographic parameters were as follows: injection and detection temperature: 250 °C and 230 °C, respectively; column oven temperature: 240 °C. The phenolic fraction was extracted in triplicate [62], and total phenol content was determined using the Folin–Ciocalteau spectrophotometric method at 750 nm [63] using a Jasco Spectrophometer (V-500, Tokyo, Japan). 

Sensory analyses were carried out using an analytical taste panel recognized by Madrid’s International Olive Oil Council (IOOC) and the Italian Ministry of Agricultural, Food, and Forestry Policies. The panel evaluated all oil samples following an incomplete randomized block design. Olive oil samples were placed in blue tasting glasses, with a temperature of 15–18 °C. A panel test was established using a standard profile sheet (IOOC/T20) modified by IBE-CNR [64]. The tasters evaluated direct or retronasal aromatic olfactory sensations (olive fruity, green/leaf, and secondary positive flavors), gustatory sensations (olive fruity, bitterness, and secondary positive flavors), and tactile/kinesthetic sensations (pungency). The tasters had to rate the intensity of the different descriptors on a continuous 0–10 cm scale. The median of sensory data and robust standard deviations were calculated. 

### 4.7. Statistical Analysis 

The data collected were elaborated using Microsoft^®^ Excel 2007/XLSTAT© (Version 2009.3.02, Addinsoft, Inc., Brooklyn, NY, USA). Significant differences were evaluated with ANOVA (*p* < 0.05), followed by Tukey’s honestly significant difference (HSD) tests.

Larval stages data were processed by applying Pearson’s chi-square test to point out significant differences in the frequencies using the “stats” package of R software v4.0.5 (Boston, MA, USA). 

## 5. Conclusions 

The results indicate that on a physiological level, the use of zeolite enriched with nitrogen increases the photosynthetic rate and drupes size, while the treatment based on zeolite alone has no effects on the photosynthetic rate as there are no differences between the values of NZ and SF. 

The BVOC characterization of both leaves and drupe emissions was carried out for the first time for the Correggiolo variety; changes in specific ratios of the BVOC blend were observed in response to the treatments: further studies currently carried out in different years could evidence a possible effect of BVOC emission changes on the plant–pathogen interactions. 

Regarding oil quality, oil produced from olives treated with NZ presented higher phenolic content, bitterness, and spiciness intensities than oils treated with EZ and SF.

Zeolite use in crop protection has considerably increased in recent years: improving the knowledge about these innovative treatments could contribute to reducing the use of insecticides, improving food quality and safety, and the sustainability of olive cultivation. 

## Figures and Tables

**Table 1:** Photosynthetic rate recorded in plants submitted to tested treatments natural zeolite (NZ), zeolite enriched with ammonium (EZ), and Spintor-Fly^®^ (SF).

Treatment	19/7	2/8	4/9	16/9	1/10
SF	14.94 ± 3.62 a	9.75 ± 2.08 b	7.53 ± 1.53 b	10.19 ± 2.05 b	9.8 ± 2.25 b
NZ	12.81 ± 1.45 b	9.53 ± 1.46 b	5.67 ± 3.08 b	10.32 ± 2.7 b	10.67 ± 1.05 ab
EZ	15.95 ± 2.5 a	12.81 ± 1.45 a	10.17 ± 2.49 a	12.54 ± 2.61 a	12.1 ± 2.57 a

Different letters in the same column indicate significant differences among means within each date of treatment application at *p* ≤ 0.05 using Tukey’s honestly significant difference (HSD) test.

**Table 2:** The total nitrogen and carbon (respectively, TN and TC) and the respective isotopic signature (δ15N and δ13C) of leaves from plants submitted to tested treatments of natural zeolite (NZ), zeolite enriched with ammonium (EZ), and Spintor-Fly^®^ (SF).

Treatment	δ13C	δ15N	TC%	TN%
SF	−26.04 ± 0.32 a	0.36 ± 0.78 ab	47.61 ± 3.68 a	1.42 ± 0.12 a
NZ	−28.79 ± 5.04 a	2.37 ± 1.24 a	50.84 ± 6.11 a	1.45 ± 0.16 a
EZ	−25.92 ± 0.37 a	−0.76 ± 0.49 b	41.5 ± 6.18 a	1.26 ± 0.24 a

Different letters in the same column indicate significant differences among means within each date of treatment application at *p* ≤ 0.05 using the HSD test.

**Table 3:** BVOC measured with GC/MS of *Olea europaea* L. cv. Correggiolo leaves submitted to tested treatments of natural zeolite (NZ), zeolite enriched with ammonium (EZ), and Spintor-Fly^®^ (SF). Data are expressed as ng m*^−^*^2^ s*^−^*^1^ ± SE.

	RT	NZ	EZ	SF
Alcohol				
2-Ethylhexanol	17.13	0.628 ± 0.217 a	0.385 ± 0.115 a	0.301 ± 0.038 a
Aldehyde				
2-Butenal	4.05	0.067 ± 0.021 a	0.035 ± 0.044 a	0.252 ± 0.195 a
Hexanal	9.32	0.041 ± 0.017 a	0.018 ± 0.003 a	0.012 ± 0.006 a
Benzaldehyde	14.38	0.084 ± 0.044 b	0.083 ± 0.013 b	0.214 ± 0.012 a
Nonanal	19.16	0.255 ± 0.114 a	0.12 ± 0.002 ab	0.026 ± 0.024 b
Decanal	21.99	0.187 ± 0.09 a	0.103 ± 0.013 ab	0.026 ± 0.009 b
Alkane				
Heptane	6.61	0.049 ± 0.03 a	0.064 ± 0.001 a	0.057 ± 0.02 a
Octane	9.84	0.107 ± 0.027 b	0.123 ± 0.022 ab	0.172 ± 0.005 a
Nonane	13.35	0.142 ± 0.053 a	0.1 ± 0.009 a	0.172 ± 0.011 a
2,2-Dimethylundecane	16.61	0.075 ± 0.042 a	0.038 ± 0.005 a	0.049 ± 0.002 a
2-Methylnonane	16.58	0.126 ± 0.041 a	0.09 ± 0.011 a	0.061 ± 0.01 a
3-Methylhexane	19.62	0.127 ± 0.042 a	0.051 ± 0.002 a	0.069 ± 0.009 a
Decane	22.37	0.139 ± 0.032 a	0.067 ± 0.015 b	0.071 ± 0.013 b
2-Methyldodecane	24.92	0.058 ± 0.033 a	0.03 ± 0.009 a	0.029 ± 0.005 a
2,3-Dimethylpentane	27.29	0.12 ± 0.041 a	0.089 ± 0.026 a	0.083 ± 0.003 a
Undecane	29.52	0.309 ± 0.086 a	0.176 ± 0.073 a	0.148 ± 0.032 a
2-Methyltridecane	33.62	0.052 ± 0.028 a	0.046 ± 0.017 a	0.047 ± 0.02 a
Arene				
Benzene	5.38	0.082 ± 0.021 a	0.062 ± 0.021 a	0.107 ± 0.018 a
Toluene	8.22	0.241 ± 0.043 ab	0.304 ± 0.036 ab	0.206 ± 0.013 b
Ethylbenzene	11.47	0.028 ± 0.006 a	0.035 ± 0.008 a	0.002 ± 0.001 b
p-Xylene	11.77	0.075 ± 0.004 a	0.08 ± 0.011 a	0.035 ± 0.007 b
o-Xylene	12.54	0.031 ± 0.006 a	0.032 ± 0.002 a	0.016 ± 0.001 b
1-Hydroxy Cumene	18.5	0.129 ± 0.038 a	0.054 ± 0.019 ab	0.028 ± 0.004 b
Ketone				
Acetophenone	17.75	0.048 ± 0.011 b	0.036 ± 0.004 b	0.17 ± 0.049 a
Aromatic Organic Compound				
Benzonitrile	15.04	nd	nd	0.381 ± 0.044
Phenol				
Phenol	15.45	0.34 ± 0.084 a	0.303 ± 0.007 a	0.235 ± 0.015 a

Acronyms are as follows: RT: retention time (min), nd: not detected, NZ: natural zeolite, EZ: zeolite enriched with NH_4_^+^, SF: Spintor-Fly^®^. Different letters in the same row indicate significant differences (*p* ≤ 0.05) according to the HSD test.

**Table 4:** BVOC measured with GC/MS of *Olea europaea* L. cv. Correggiolo fruits submitted to tested treatments of natural zeolite (NZ), zeolite enriched with ammonium (EZ), and Spintor-Fly^®^ (SF). Data are expressed as ng m^−2^ s^−1^ ± SE.

		First Collection			Second Collection		
	RT	NZ	EZ	SF	NZ	EZ	SF
Alcohol							
2-Propanol	3.57	0.039 ± 0.006 b	0.027 ± 0.007 b	0.058 ± 0.003 b	0.049 ± 0.011 b	0.053 ± 0.015 b	0.086 ± 0.007 a
Aldehyde							
Hexanal	9.11	0.007 ± 0.001 c	0.007 ± 0.001 c	0.01 ± 0.001 c	0.022 ± 0.001 a	0.012 ± 0.001 b	0.007 ± 0.001 c
Benzaldehyde	14.33	0.007 ± 0.002 b	0.006 ± 0.002 b	0.021 ± 0.008 a	0.008 ± 0.001 b	0.007 ± 0.001 b	0.004 ± 0.001 b
Octanal	16.07	tr	0.003 ± 0.001 a	0.002 ± 0.001 b	0.003 ± 0.001 a	tr	tr
Nonanal	19.09	0.008 ± 0.001 b	0.011 ± 0.001 b	0.015 ± 0.001 b	0.045 ± 0.004 a	0.014 ± 0.001 b	0.017 ± 0.001 b
Decanal	21.94	tr	0.002 ± 0.001	0.003 ± 0.001 a	0.002 ± 0.001 a	nd	tr
Alkane							
Octane	9.78	0.021 ± 0.001 a	0.012 ± 0.003 a	0.027 ± 0.009 a	0.031 ± 0.006 a	0.012 ± 0.001 a	0.021 ± 0.002 a
Nonane	13.29	0.004 ± 0.001 a	nd	0.003 ± 0.001 a	0.002 ± 0.001 a	nd	nd
Undecane	27.23	0.004 ± 0.001 c	0.005 ± 0.001 b	0.007 ± 0.001 a	0.002 ± 0.001 d	nd d	0.002 ± 0.001 d
Arene							
Benzene	5.34	0.002 ± 0.001 b	nd	0.003 ± 0.001 b	0.003 ± 0.001 b	0.006 ± 0.001 a	0.006 ± 0.001 a
Toluene	8.18	0.003 ± 0.001 a	0.002 ± 0.001 a	0.005 ± 0.003 a	0.004 ± 0.001 a	0.004 ± 0.001 a	0.005 ± 0.001 a
Ethylbenzene	11.39	0.003 ± 0.001 b	nd	0.003 ± 0.002 b	0.003 ± 0.001 b	0.003 ± 0.001 b	0.006 ± 0.002 a
p-Xylene	11.71	0.003 ± 0.001 a	nd	0.005 ± 0.003 a	0.003 ± 0.001 a	0.002 ± 0.001 a	0.006 ± 0.002 a
o-Xylene	12.48	tr	tr	tr	tr	tr	tr
Ester							
*cis*-3 Hexenyl Acetate	16.28	0.003 ± 0.001 b	0.005 ± 0.002 a	0.003 ± 0.001 b	0.003 ± 0.001 a	0.002 ± 0.001 b	tr
Ether							
Ethyl Ether	3.65	0.082 ± 0.033 a	0.027 ± 0.013 a	0.04 ± 0.008 a	0.03 ± 0.016 a	0.018 ± 0.009 a	0.17 ± 0.074 a
Ketone							
2-Propanone	3.54	0.092 ± 0.026 b	0.061 ± 0.01 b	0.177 ± 0.025 a	0.081 ± 0.011 b	0.08 ± 0.027 b	0.112 ± 0.003 b
2-Pentanone	5.94	0.019 ± 0.002 b	nd	0.013 ± 0.006 b	0.008 ± 0.001 b	0.007 ± 0.002 b	0.032 ± 0.003 a
Monoterpenoid							
Citronellol	31.96	nd	0.001 ± 0.001	0.006 ± 0.004	nd	nd	nd
Sesquiterpene							
α-Copaene	26.75	nd	nd	0.001 ± 0.001 a	0.002 ± 0.001 a	0.003 ± 0.001 a	0.001 ± 0.001 a
Furan							
Tetrahydrofuran	4.76	0.048 ± 0.009 b	0.041 ± 0.01 b	0.064 ± 0.011 b	0.082 ± 0.035 b	0.054 ± 0.019 b	0.268 ± 0.106 a
Aromatic Organic Compound							
Benzonitrile	15	tr	0.004 ± 0.001	0.004 ± 0.002	0.004 ± 0.001	tr	tr
Phenol							
Phenol	15.41	tr	tr	0.006 ± 0.005 a	tr	tr	nd

Acronyms are as follows: RT: retention time (min), nd: not detected, tr: trace, NZ: natural zeolite, EZ: zeolite enriched with NH4^+^, SF: Spintor-Fly^®^. Different letters in the same row indicate significant differences (*p* ≤ 0.05) according to the HSD test.

**Table 5:** Quality index of oil samples cv. Correggiolo submitted to tested treatments of natural zeolite (NZ), zeolite enriched with ammonium (EZ), and Spintor-Fly^®^ (SF).

Treatment	Free Acidity ^1^	Peroxid Number ^2^	k232	k270	C16	C16:1	C18	C18:1	C18:2	C18:3
SF	0.13	8.00	1.86	0.11	13.89	1.26	2.24	72.97	8.35	0.51
NZ	0.14	5.60	2.17	0.17	13.77	1.29	2.35	73.74	7.53	0.56
EZ	0.13	8.30	1.78	0.10	13.77	1.33	2.05	73.59	7.95	0.52

^1^ Free acidity is expressed as g/100 g of oleic acid; ^2^ peroxide number as mEq O_2_ kg^−1^ oil; Fatty acids compotition as g/100 g of total fat.

**Table 6:** Sensory attributes of olive oils produced by plants treated with natural zeolite (NZ), zeolite enriched with ammonium (EZ), and Spintor-Fly^®^ (SF).

Treatment	Olfactory Olive Fruity	Olfactory Pleasant Flavors	Gustatory Olive Fruity	Bitter	Pungent	Grass	Gustatory Pleasant Flavors
SF	4.9	3.4	3.7	3.5 b	3.7 b	1.5 b	1.4
NZ	5.5	3.6	5.1	5.6 a	5.3 a	3.3 a	3.0
EZ	4.9	3.4	4.5	3.8 b	4.4 ab	2.8 ab	2.5

Different letters in the same column indicate significant differences (*p* < 0.05) according to the HSD test.

## Data Availability

All data, tables, and figures in this manuscript are original. Raw data of this experiment is fully available upon request to the corresponding author.

## Supplementary Materials

The following supporting information can be downloaded at https://www.mdpi.com/article/10.3390/plants13050698/s1, Table S1: Element characterization of soil samples. Treatments: natural zeolite (NZ), zeolite enriched with ammonium (EZ), and Spintor-Fly^®^ (SF). Different letters in the same column indicate significant differences (*p* < 0.05) according to the HSD test; Table S2； Element characterization of leaves submitted to tested treatments natural zeolite (NZ), zeolite enriched with ammonium (EZ), and Spintor-Fly^®^ (SF). Different letters in the same column indicate significant differences (*p* < 0.05) according to the HSD test.
